# A Mobile Phone Food Record App to Digitally Capture Dietary Intake for Adolescents in a Free-Living Environment: Usability Study

**DOI:** 10.2196/mhealth.3324

**Published:** 2015-03-13

**Authors:** Shanon L Casperson, Jared Sieling, Jon Moon, LuAnn Johnson, James N Roemmich, Leah Whigham

**Affiliations:** ^1^Grand Forks Human Nutrition Research CenterAgricultural Research ServiceUnited States Department of AgricultureGrand Forks, NDUnited States; ^2^MEI Research, Ltd.Edina, MNUnited States; ^3^Paso del Norte Institute for Healthy LivingEl Paso, TXUnited States

**Keywords:** adolescents, dietary food records, smartphone app, dietary assessment, food record app

## Abstract

**Background:**

Mobile technologies are emerging as valuable tools to collect and assess dietary intake. Adolescents readily accept and adopt new technologies; thus, a food record app (FRapp) may be a useful tool to better understand adolescents’ dietary intake and eating patterns.

**Objective:**

We sought to determine the amenability of adolescents, in a free-living environment with minimal parental input, to use the FRapp to record their dietary intake.

**Methods:**

Eighteen community-dwelling adolescents (11-14 years) received detailed instructions to record their dietary intake for 3-7 days using the FRapp. Participants were instructed to capture before and after images of all foods and beverages consumed and to include a fiducial marker in the image. Participants were also asked to provide text descriptors including amount and type of all foods and beverages consumed.

**Results:**

Eight of 18 participants were able to follow all instructions: included pre- and post-meal images, a fiducial marker, and a text descriptor and collected diet records on 2 weekdays and 1 weekend day. Dietary intake was recorded on average for 3.2 (SD 1.3 days; 68% weekdays and 32% weekend days) with an average of 2.2 (SD 1.1) eating events per day per participant. A total of 143 eating events were recorded, of which 109 had at least one associated image and 34 were recorded with text only. Of the 109 eating events with images, 66 included all foods, beverages and a fiducial marker and 44 included both a pre- and post-meal image. Text was included with 78 of the captured images. Of the meals recorded, 36, 33, 35, and 39 were breakfasts, lunches, dinners, and snacks, respectively.

**Conclusions:**

These data suggest that mobile devices equipped with an app to record dietary intake will be used by adolescents in a free-living environment; however, a minority of participants followed all directions. User-friendly mobile food record apps may increase participant amenability, increasing our understanding of adolescent dietary intake and eating patterns. To improve data collection, the FRapp should deliver prompts for tasks, such as capturing images before and after each eating event, including the fiducial marker in the image, providing complete and accurate text information, and ensuring all eating events are recorded and should be customizable to individuals and to different situations.

**Trial Registration:**

Clinicaltrials.gov NCT01803997. http://clinicaltrials.gov/ct2/show/NCT01803997 (Archived at: http://www.webcitation.org/6WiV1vxoR).

## Introduction

Assessing dietary intake accurately is imperative to understanding the impact it has on health risk and developing intervention programs. Unfortunately, obtaining dietary intake records from adolescents is difficult at best, as their eating behaviors are influenced by a complex interaction of sociological, psychological, and biological factors [1-3]. Self-image, sporadic eating (meal skipping, random snacking, school events that interfere with scheduled meals), frequently eating outside the home, and the complexity of our food supply add to the difficulty of obtaining an accurate assessment of adolescent dietary intake [1,4,5]. More importantly, Goodwin et al [6] reported adolescents as being reluctant to record their dietary intake via the traditional pen and paper method, especially around peers, “for fear of what they would say.” Furthermore, these adolescents were reported as saying that they would alter what they ate to simplify their dietary recording.

Mobile technologies are emerging as a valuable tool to assist in the collection and assessment of dietary intake [7-17]. Approximately 75% of American adolescents carry some form of mobile technology on their person at all times (eg, iPod, mobile phone); hence a mobile youth culture has emerged [18]. Compared to adults, adolescents are more confident and efficient at using mobile technologies and have expressed a preference toward assessment methods that use technology [7,8]. Accordingly, employing readily available technology that is incorporated easily into their lifestyle may help involve adolescents in dietary intake assessment and increase reporting amenability and accuracy. Indeed, when asked to capture images of foods consumed for one day, adolescents (11-15 years) went so far as to capture images of single food items [7].

Methodological advancements have been made in image-based dietary assessment tools that use mobile telephone native technologies (eg, camera, Wi-Fi). With images of foods, beverages, and a standard reference object of known size (fiducial marker) captured before and after an eating event, portion sizes can be estimated and completeness of the record can be evaluated by trained individuals and computer programs, providing a more accurate assessment of dietary intake [9,12]. In adolescents, mobile technologies have been used under controlled conditions to aid in method development and to determine user proficiency [7-9,13]. Therefore, we sought to determine the amenability of adolescents, in a free-living environment, to use a mobile phone food record app (FRapp) to record their dietary intake.

## Methods

### Participants

We collected data from a convenience sample of healthy, community-dwelling adolescents (n=18) participating in an after school program. The only inclusion criterion was grade level (6th, 7th, or 8th); there were no exclusion criteria. The project was approved by the Institutional Review Board of the University of North Dakota. Written informed assent and consent were obtained prior to participation from the adolescent participants and their parents, respectively. Participants were recruited during an open house at a local middle school. School administrators approved of the project and students were given permission to use the study mobile phones during school. Participants were instructed to continue all regular activities of daily living and maintain their usual diet. Height was measured in duplicate to the nearest 0.1 cm using a stadiometer. Body weight was measured using a calibrated digital scale to the nearest 0.1 kg. Parents and participants completed a demographic form regarding race, ethnicity, birth date, age, sex, and number of people living in the household. The participants also were asked their grade level.

### Experimental Protocol

Participants were assigned a mobile phone (Motorola Defy, Motorola Mobility LLC.) with the FRapp (ActiPal; MEI Research, LTD) installed. Mobile phones did not have active cellular contracts to ensure compliance with school regulations but had internet access when Wi-Fi was available. We asked parents to not involve themselves when their child recorded dietary intake because we wanted to determine the quality and quantity of data that the adolescent would provide.

**Figure 1:**
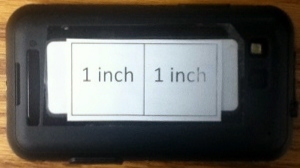
Fiducial marker.

**Figure 2:**
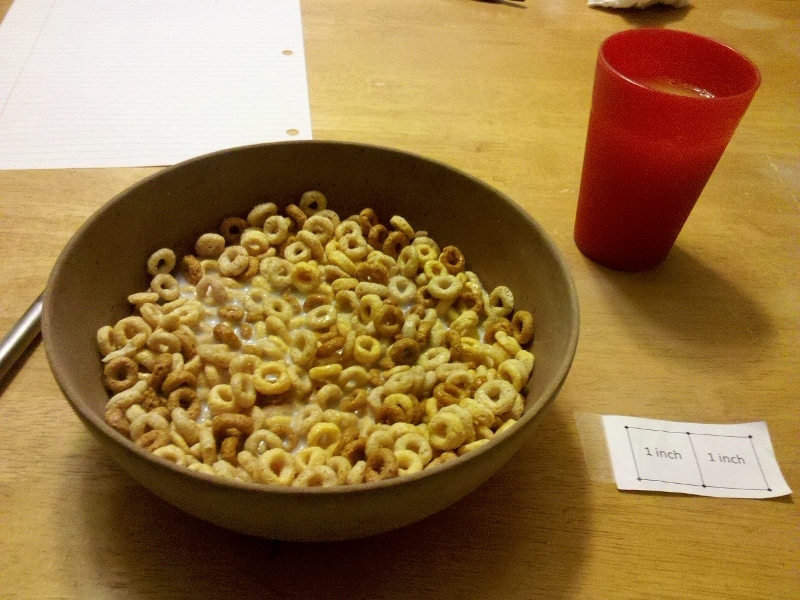
Fiducial marker with food.

Participants received a one-on-one demonstration on how to use the FRapp, including where to place the fiducial marker and how to hold the mobile phone when capturing images to achieve the requested 45° angle. A fiducial marker is needed in all captured images to serve as a size reference. The fiducial marker was 2 inches by 1 inch and printed on standard white paper (Figure 1). Participants practiced capturing images of simulated food items and entering text descriptions until they were comfortable using the FRapp. Participants were asked to capture before and after images of all foods and beverages consumed, along with the supplied fiducial marker (Figure 2) and to enter text descriptions that provided as much detail as possible about the foods and beverages consumed using the FRapp for 3-7 days, ensuring that at least 2 of the days were during the week and 1 was a weekend day. Text descriptions were to include descriptors (eg, 2% milk, low-fat yogurt) and estimated amounts (eg, 1 carton, 2 cups, 3 ounces). When at home, participants were asked to measure the amount of food they were planning on eating using kitchen scales and measuring implements if possible (kitchen scales and measuring implements were not provided).

### Food Record App

The FRapp was designed for natural settings using smartphone technologies. Unlike previous apps and single-purpose electronic devices to record dietary intake [7,9,10,13,17,19], the FRapp integrated not only the camera function but also text entry, prompts predefined for eating occasions (eg, breakfast, lunch, dinner, snack), and real-time communication between user and clinician/researcher (when connected via cellular contract or Wi-Fi, not used in this study). It is important to note that the FRapp could be used in numerous ways. There are six dietary intake input methods that can be enabled within the FRapp by the investigator: (1) capturing and annotating meal images, (2) typing in free text food descriptors, (3) speech-to-text conversions with food item extraction, (4) record voice for later playback, (5) capturing food label/nutrition facts/barcode photos, and (6) selecting from recently consumed food sets. These input methods can be used singly or in combination by participants [20]. For this study, we employed capturing and annotating meal images and typing in free text food descriptors. We felt that using only two input methods would minimize any participant apprehension and increase amenability to using the FRapp.

### Food Record Input

**Figure 3:**
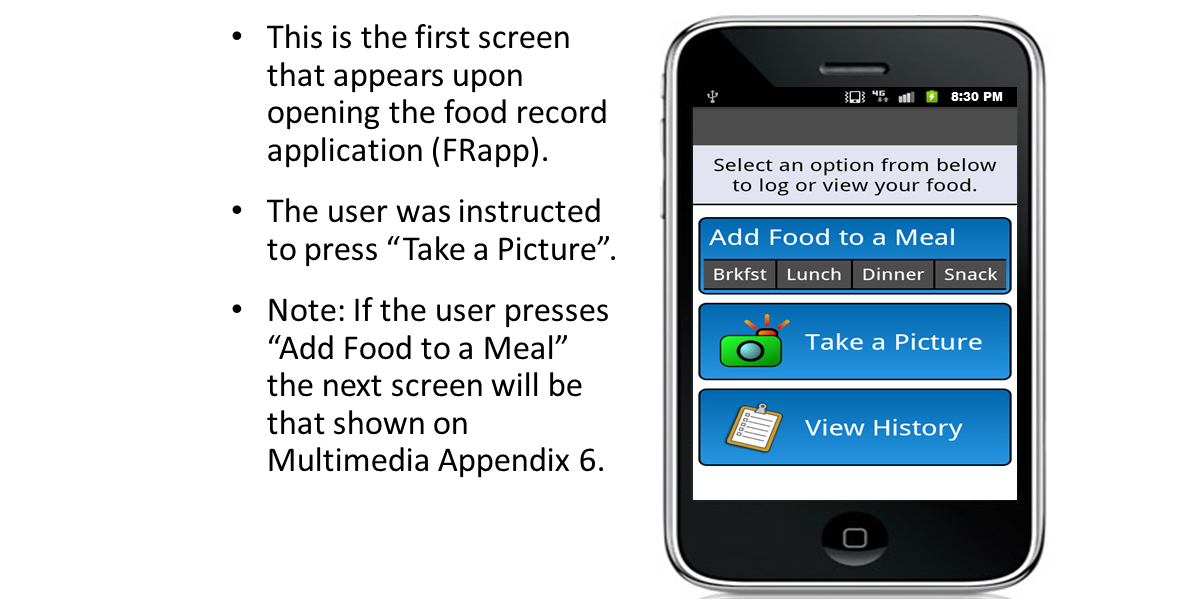
Initialization of the FRapp.

**Figure 4:**
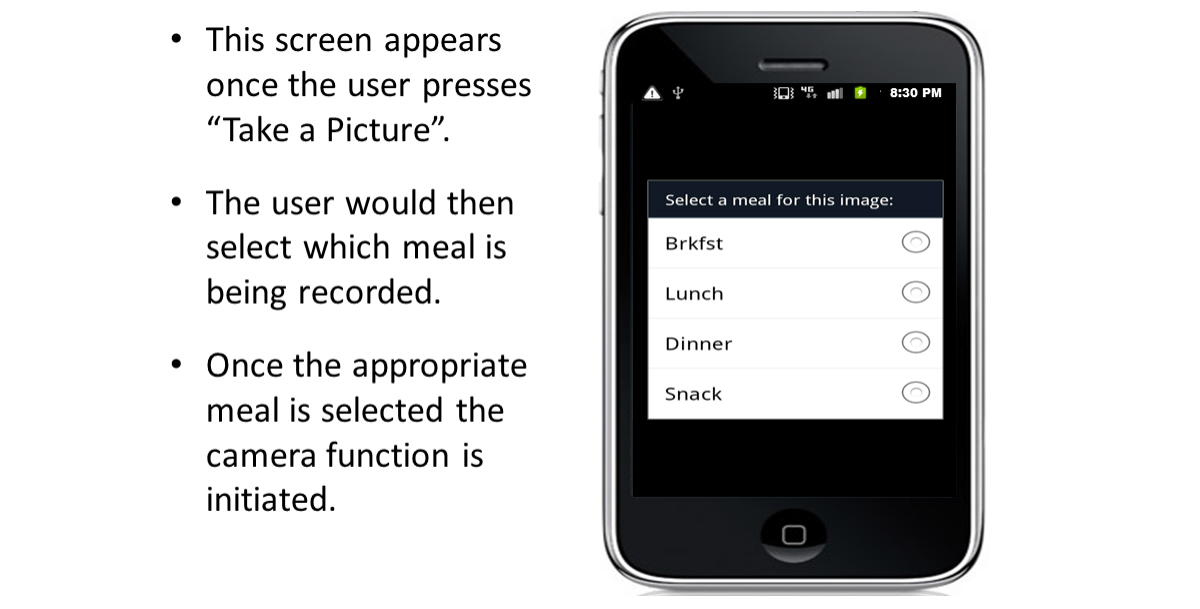
Identification of the eating event that is going to be recorded.

**Figure 5:**
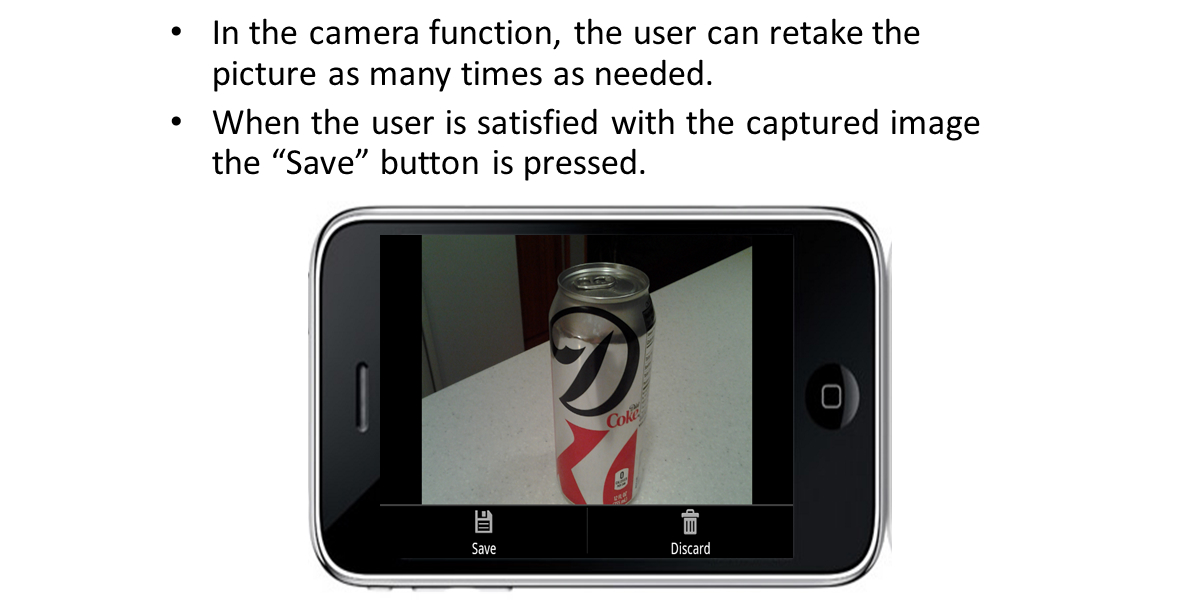
Using the camera function to digitally capture food and beverage images.

**Figure 6:**
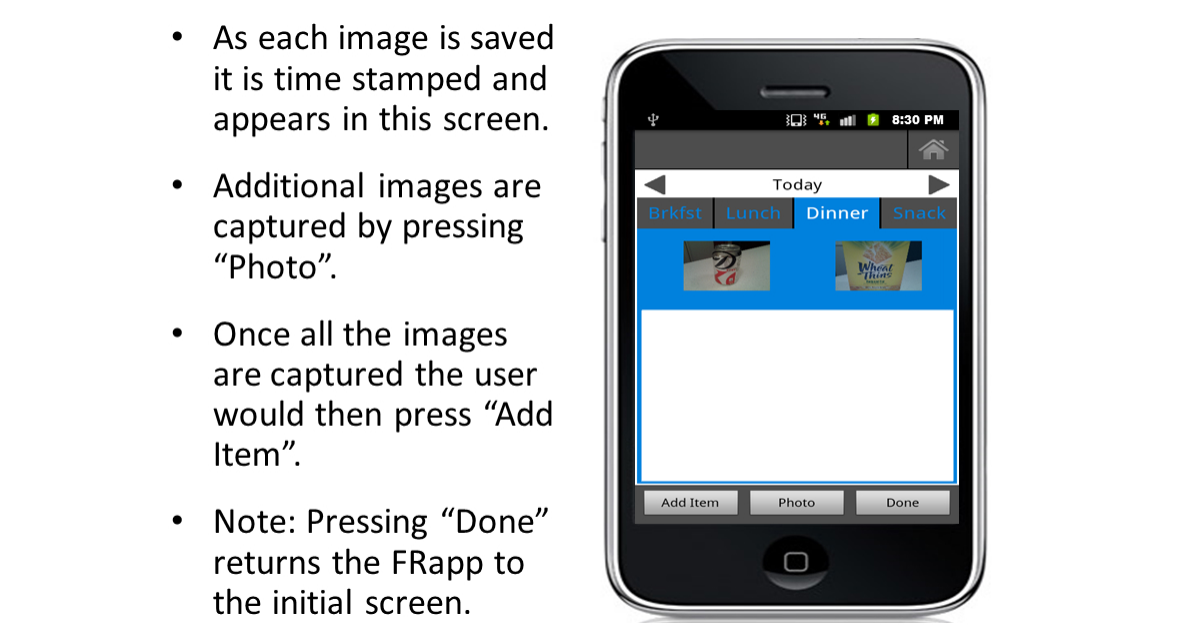
All images for the eating event are displayed.

The FRapp guided participants through the process we configured to enter their daily dietary intake. When the FRapp was first opened, the participant was asked if they wanted to Add Food to a Meal, Take a Picture, or View History (Figure 3). During the practice session, participants were shown how to select the Take a Picture option. After selecting this option, the FRapp required the participant to select a meal occasion. After the participant selected breakfast, lunch, dinner or snack (Figure 4), the camera function initiated automatically. We instructed the participants to hold the phone at a 45° angle and to confirm that all foods and beverages plus the supplied fiducial marker were visible on the mobile phone screen before capturing the image. If the fiducial marker was not available (left at home or misplaced), the participants were instructed to use an object of a known/predictable size (eg, coin, ballpoint pen). The image then was displayed for review and the user was given the choice to retake, cancel, or save the current image (Figure 5). Each participant was asked to verify again that all items were captured in the image before saving. The participants were allowed to retake images as many times as needed. Once the participants were satisfied with the images, they pressed Save and the image was time-stamped and securely uploaded to a data storage server to be viewed in real-time (when connected via cellular contract or Wi-Fi) or analyzed later. The image(s) was then displayed on the screen in the meal tab that had been chosen before the image was captured (Figure 6).

**Figure 7:**
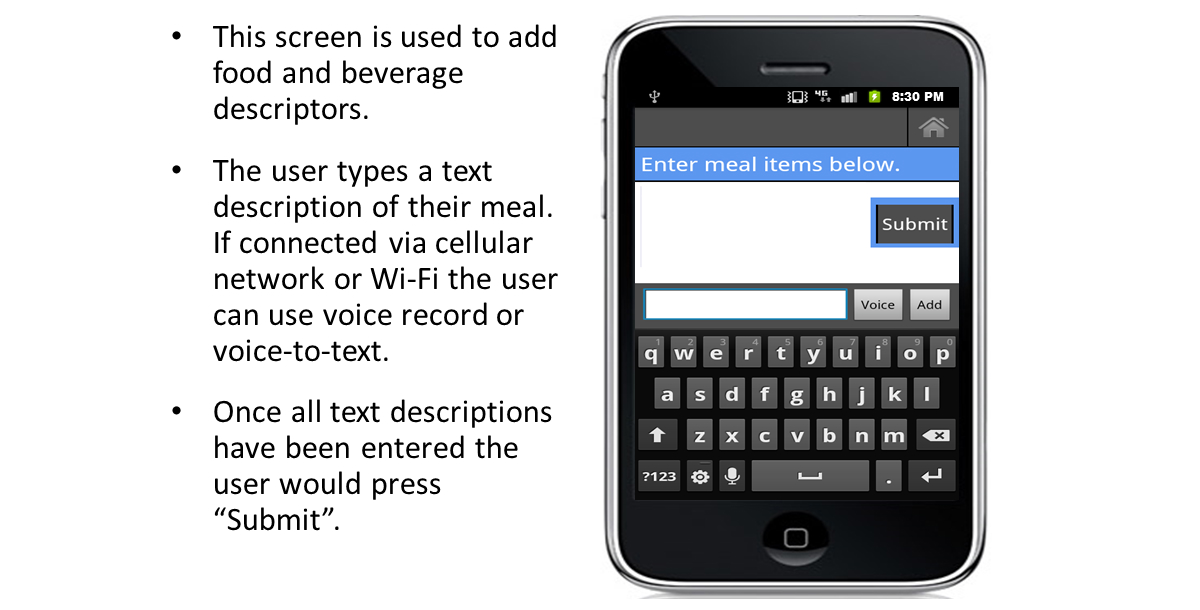
Text entry of all food/beverage descriptions.

**Figure 8:**
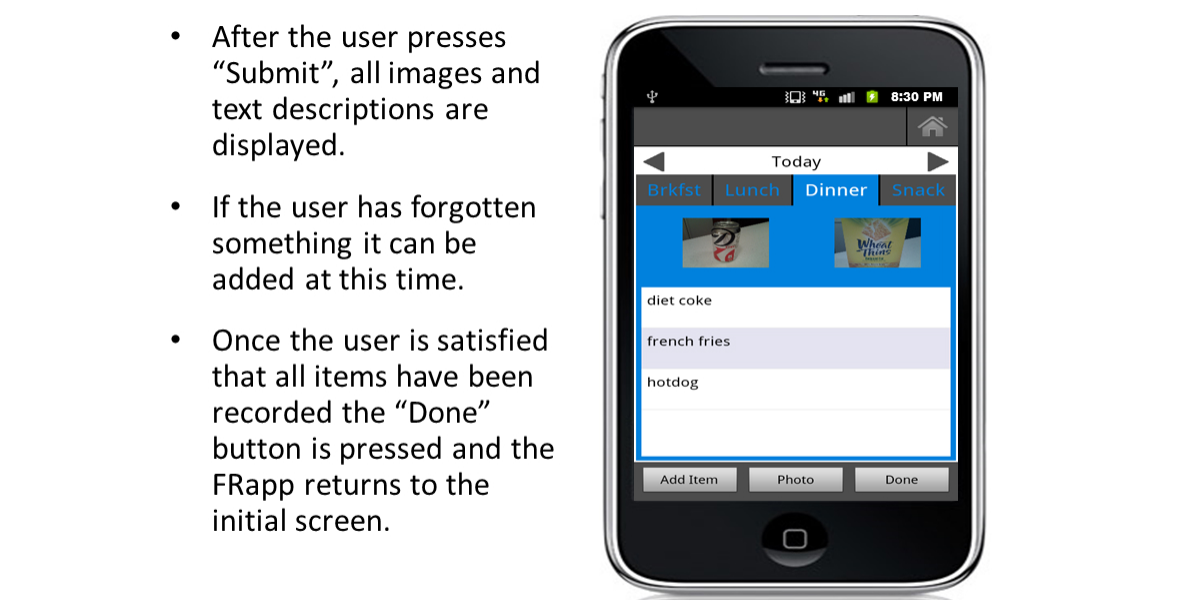
Display of everything entered for each eating event.

After capturing meal images, the participants were asked to record information about the food and beverage items. We instructed the participant to tap on the Add Item button at the bottom of the screen which would display a keyboard (Figure 7). The participant would then enter a description about each food and beverage item, including type (eg, 2% milk, Diet Sprite, light ranch dressing) and amount (eg, 1 cup, 1 can, 6 ounces). After annotating each item, the participant pressed Add and the text description was displayed under the captured image. The participant was asked to review all items and make any necessary corrections before pressing Submit. The images and food items were then shown under each meal tab (Figure 8). Participants were asked to capture an image of their eating utensils with any leftover food and beverage when they were finished eating. After capturing pre- and post-meal images and entering all text descriptions, the participant was asked to carefully review all items entered before pressing Done and exiting the FRapp. All data were uploaded to a secure data storage server and accessed through a management website (PiLR Healthware, MEI Research).

### Image and Text Description Evaluation

Amenability was assessed by how well participants followed the instructions to capture meal images (ie, all foods, beverages, and a fiducial marker are shown in the image; pre- and post-meal images are captured), provide text descriptions, and record a minimum of 3 days of dietary intake (2 weekdays and 1 weekend day). Images were coded as pre-meal only if no post-meal image was captured, post-meal if no pre-meal image was captured, and pre/post if both images were captured. Images were then compared to corresponding text descriptions to determine if all items were included. Images were coded as complete if all foods and beverages available for consumption during the eating event, a fiducial marker, and the eating utensils were completely visible. Images were coded as incomplete if any item was missing or not completely visible. In addition, the image was coded as yes if a text description was provided or no if no text description was proved. If no image was captured, the text description was coded as text only.

### Statistical Analysis

The numbers of captured images of meals and snacks along with text descriptions were evaluated with frequencies. A log-linear model was fit to the frequency counts to test whether the inclusion of a text description was associated with the completeness of the captured images or if post-meal images were captured. The Glimmix procedure in SAS V9.3 (SAS Institute, Inc.) was used to fit the models.

## Results

Participant characteristics are presented in Table 1. Dietary intake was recorded on average for 3.2 (SD 1.3) days (68% weekdays and 32% weekend days) with an average of 2.2 (SD 1.1) eating events per day per participant. Twelve of the 18 participants collected dietary intake records for at least one meal on 3 or more separate days. Eight participants followed all directions, assuming that breakfast, lunch, or dinner meals with no data entry were not consumed by the participants, 3 recorded dietary intakes on 2 weekend days and 1 weekday, and 1 recorded dietary intake only on weekdays. Eight participants recorded an eating event for breakfast, lunch and dinner on the same day. It is not known if meals were skipped or if a participant forgot to record eating events. On weekdays the evening meal was the primary eating event with no recorded entry and on weekend days the morning and mid-day meals were the primary eating events with no recorded entry.

**Table 1:** Subject characteristics. Anthropometric data are presented as mean (SD). All other data are presented as the total number of participants for each category.

		Males (n=5)	Females (n=13)
Age, y		11.8 (1.1)	11.2 (0.7)
Weight, kg		45.4 (9.9)	57.7 (22.9)
Height, cm		150.2 (8.4)	154.0 (10.3)
Body mass index (BMI), kg/m^2^		19.9 (2.6)	23.7 (7.1)
**BMI, percentile**			
	≥95th		5
	85th-94th	1	2
	5th-84th	4	6
**Race/ethnicity**			
	Non-Hispanic White	5	6
	American Indian/Alaska Native		6
	African American		1

A total of 143 eating events were recorded: 36, 33, 35, and 39 were breakfasts, lunches, dinners, and snacks, respectively. One participant recorded all eating events (10 total on 5 different days) as snacks. Images were captured for 109 eating events, and only a text description was provided for an additional 34. Of the 109 eating events with captured images, 23 included a pre- and post-meal image, a fiducial marker, and a text description. Table 2 summarizes the recorded eating events.

Of the 109 eating events with captured images, 66 of the images were coded as complete (all foods and beverages available for consumption during the eating event, a fiducial marker, and the eating utensils were completely visible). While the participants were provided with a fiducial marker, the instructions also stated that they could use a standard object of known/predictable size. Thirteen participants used the supplied fiducial marker for at least one eating event. The supplied fiducial marker was used for 36 eating events while an object of known size (for this study eyeglasses and click-top ballpoint pens) were used for 30 eating events coded as complete.

Participants had difficulties remembering to capture a post-meal image. Of the 109 eating events with captured images, only 44 post-meal images were captured. When asked, participants simply stated that they forgot or that they didn’t think we wanted a picture of a dirty plate. Notably, when a post-meal image was captured, 32 included a complete place setting with a fiducial marker.

Thirteen participants provided text descriptors although only 5 attempted to report the amount consumed. A greater (*P*<.001) number of eating events included a text description than did not. Text was provided for a total of 112 eating events of which 78 were paired with a captured image.

For this project only 3 participants recorded eating events only at home, whereas 15 recorded a total of 28 eating events outside the home. Ten participants recorded eating events outside the home only at school, 3 recorded eating events outside the home only at restaurants/fast-food establishments, and 2 recorded eating events outside the home at both school and restaurants/fast-food establishments. Of the 28 eating events outside the home, 21 (75%) were recorded at school (5 breakfasts and 16 lunches) and 7 (25%) were recorded at a restaurant.

**Table 2:** Evaluation of the dietary intake records provided through FRapp usage.

			Eating event (n=143)			
Image	Completeness	Text included	Breakfast n (%)	Lunch n (%)	Dinner n (%)	Snack n (%)
Pre/post- meal images captured	Complete	Yes	6 (4.2)	5 (3.5)	8 (5.6)	4 (2.8)
		No	4 (2.8)	3 (2.1)	0 (0.0)	0 (0.0)
	Incomplete	Yes	4 (2.8)	2 (1.4)	1 (0.7)	2 (1.4)
		No	1 (0.7)	2 (1.4)	2 (1.4)	1 (0.7)
Pre-meal only images captured	Complete	Yes	5 (3.5)	0 (0.0)	5 (3.5)	16 (11.2)
		No	4 (2.8)	2 (1.4)	1 (0.7)	3 (2.1)
	Incomplete	Yes	1 (0.7)	6 (4.2)	7 (4.9)	5 (3.5)
		No	2 (1.4)	2 (1.4)	0 (0.0)	3 (2.1)
Post-meal only images captured	Complete	Yes	0 (0.0)	0 (0.0)	0 (0.0)	0 (0.0)
		No	0 (0.0)	0 (0.0)	0 (0.0)	0 (0.0)
	Incomplete	Yes	0 (0.0)	1 (0.7)	0 (0.0)	0 (0.0)
		No	0 (0.0)	1 (0.7)	0 (0.0)	0 (0.0)
None	Text only	Yes	9 (6.3)	9 (6.3)	11 (7.7)	5 (3.5)

## Discussion

### Principal Findings

Our goal was to determine the amenability of adolescents, in a free-living environment with minimal parental input, to use the FRapp to record their dietary intake. The data from this study support the use of a FRapp to assist in the collection of adolescent dietary intake records, as the adolescents did a reasonable job of following instructions to capture meal images, provide text descriptions, and record a minimum of 3 days of dietary intake.

Any tool that provides a simple means to improve adolescent amenability with collecting dietary information without changing usual eating behaviors is an important advance. Compared to the traditional pen and paper method of collecting dietary intake records, the utilization of captured images provides similar [9] or improved reporting accuracy [19] in children and adolescents under controlled, supervised conditions in the home setting. In a study by Higgins et al [9], 50% of the participants provided a complete 3-day photographic food record while the other 50% had missing images (ie, no images or missing either the pre- or post-meal image). Nonetheless, they reported no difference in energy intake between photographic food records and a 3-day food diary. In a study by Svensson et al, participants captured images for 65% of their recorded foods using a digital camera, resulting in a more accurate estimate of dietary intake (24% underestimation of energy intake) [19] compared to the traditional pen and paper food record alone (35% and 41% underestimation of energy intake) [21,22]. It is important to note that in these 2 studies parents played a significant role in recording the adolescent’s dietary intake, and the captured images were used to assist in preparing traditional pen and paper dietary intake records [9,19]. Unique from previous studies, the FRapp was used as a standalone tool for the collection of dietary intake with minimal parental input. Adolescents have great autonomy of use of cell phones so this study was designed to test their amenability to use a FRapp without parent supervision and in a less controlled free-living environment. Dietary intake information was collected on 143 eating events with 44% of the participants recording dietary intake for the standard 2 weekdays and 1 weekend day and an additional 17% recording dietary intakes on 2 weekend days and 1 weekday.

A primary concern with using mobile technologies to record adolescent dietary intake is capturing all dietary intake. For the current project, dietary intake was recorded on average for 2.2 (SD 1.1) eating events per day per participant. It is important to determine whether meals were skipped or the participant forgot to record an eating event. To address the issue of low report rates while attempting to keep participant burden low, an additional prompt will be added to the FRapp. Upon initializing the FRapp, the participant would be asked “Did you eat and/or drink anything since your last entry at [time] on [date]?” The participant would have to answer yes or no before the FRapp would progress to the next screen. If the participant answers yes, the keyboard will be displayed for the participant to record a description about each meal for which they forgot to capture images (when connected via an active calling plan or Wi-Fi, voice recording or voice-to-text could be used). This prompt would elicit participant recall and possibly reduce the number of eating events not being recorded due to forgetfulness.

Another concern with using mobile technologies to record adolescent dietary intake is capturing both a pre-meal image that includes all foods and beverages and a post-meal image showing what was consumed. This includes capturing before and after images for all additional food servings (second and third helpings). Without these images, dietary intake cannot accurately be determined. In the current project, the majority (61%) of images captured included all foods and beverages that were known to be consumed during that particular eating event. Nonetheless, remembering to capture a post-meal image was problematic. These results are in line with previous reports [8,13] and support the need for delivery of triggers and prompts to remind participants to capture images before and after each meal. One example of an automated trigger is a simple timing function that would initiate a sound and/or vibration after a specified duration following an event [23]. The timing of the trigger would be customized to each participant.

A concern in evaluating adolescents’ electronic dietary food intake images is the inclusion of a fiducial marker [8,13]. Daugherty, et al [8] found that a large fiducial maker (approximately 4× 4 inches) was difficult to include without being partially concealed. Six, et al [13] found that adolescents had a difficult time including a USB flash drive-sized fiducial marker. In both studies adolescents stated that a marker the size of a credit card might be easier to carry and use. To address this issue we supplied a fiducial marker (Figure 1) that would adhere to the back of the smartphone and be easily removed and reapplied to decrease the burden of carrying an additional item and to serve as a reminder to include the marker in the image. We found that 13 of the 18 participants used the supplied fiducial marker. Overall, 61% of the captured images included a marker that could be easily used to estimate portion sizes. Designing a fiducial marker that is easily remembered and used by adolescents remains an important consideration. A notification that appears on the screen asking the participant if the marker is in place before allowing the image to be captured may be helpful to ensure that a marker is included in every captured image. Perhaps a more viable solution for adolescents would be an electronic fiducial marker that is applied automatically when the camera function is active. The camera pixels subtended by an object would then be used to register size, thereby forgoing the need to remember to include an external fiducial marker in the image.

While captured images increase the accuracy of estimated portion sizes [11], identification of some food and beverage items (eg, mineral water vs a clear sugary carbonated beverage, different brands and types of cooked cereals, condiments on a sandwich) proved to be challenging. Participants were asked to provide a text description of all food and beverage items to be consumed, including amounts, to avoid misinterpretation of the items in the captured images. Text descriptions were then compared to the accompanied captured image. On several occasions the text descriptions were needed to identify the food item (eg, twice baked potatoes, Sprite). Furthermore, the text descriptions provided details on foods not visible in the image. While informative as to what was consumed, the text descriptions did not fully quantify the food item. Some participants recorded the number of food items consumed (ie, 2 chicken strips) but not the amount (ie, 4 oz. chicken strip, 2 tbsp. peanut butter on toast). In contrast, when only the text description was provided there was no way to determine the accuracy of the information. Therefore, using both images and text descriptions is necessary to optimize assessment of dietary intake.

### Limitations

A limitation of the present study was the use of the FRapp as a stand-alone tool to collect adolescent dietary intake. Only 16% of recorded eating events using the FRapp included the necessary components of pre- and post-meal images, a fiducial marker, and text descriptions of the foods. Missed meal images cannot be recorded in retrospect; however, the information can be retrospectively recorded via text or by voice recording. Moreover, a live connection with study personnel would allow for real-time follow-up when data are missing. The use of set meal designations may have increased variability. For example, drinking a caloric beverage may not have been recorded because the participant did not consume it during a meal and may not have regarded it as a snack. This limitation would apply whether the recording method was electronic or the more traditional paper and pencil method. If researchers have no need to determine meal designation, the FRapp can be adapted to collect intake data without a meal designation. A follow-up interview by trained staff is a valuable methodological addition to increase the likelihood of collecting complete and accurate dietary intake from all age groups. Nevertheless, capturing meal images, either pre-meal only or pre- and post-meal, will offer an additional source of dietary intake verification and strengthen records.

### Conclusion

The FRapp was used by adolescents in a variety of settings, although they were not amenable to following all of the directions. Giving investigators and study participants flexibility to employ a dietary intake recording method that is most suited to their needs will maximize the accuracy, quality, and completeness of the data. In addition, when used with an active cellular contract or Wi-Fi, real-time monitoring by research or clinical staff can provide individual customized prompts (ie, text messages, emoticons) when items in the image are not fully visible, the image is blurred, or post-meal images have not been received within a specified time period. Results from this project provide direction for further mobile phone app development to maximize the quality and completeness of adolescent dietary intake records.

## Abbreviations

FRapp: food record app
